# C-reactive protein conformations and their association with the IL-1β/IL-6 pathway in ocular inflammatory conditions

**DOI:** 10.3389/fimmu.2025.1601145

**Published:** 2025-05-12

**Authors:** Mercedes S. Nabaes Jodar, Víctor Llorenç, Marc Figueras-Roca, Maite Sainz de-la-Maza, Alfredo Adán, Blanca Molins

**Affiliations:** ^1^ Group of Ocular Inflammation, Clinical and Experimental Studies, Institut d’Investigacions Biomèdiques Agustí Pi i Sunyer (IDIBAPS), Barcelona, Spain; ^2^ Institut Clínic d’Oftalmologia (ICOF), Hospital Clínic de Barcelona, Barcelona, Spain

**Keywords:** C-reactive protein (CRP), interleukin-1β (IL-1β), interleukin-6 (IL-6), ocular inflammation, uveitis, diabetic macular edema

## Abstract

**Introduction:**

C-reactive protein (CRP) plays a critical role in the innate immune system and serves as a biomarker for various inflammatory conditions. CRP is a dynamic protein undergoing conformational changes between pentameric (pCRP) and monomeric (mCRP) conformations. pCRP is the well-established systemic marker of inflammation, while mCRP is associated with localized tissue inflammation.

**Methods:**

This study aimed to evaluate systemic levels of pCRP, mCRP, interleukin-6 (IL-6), and interleukin-1β (IL-1β) in patients with a variety of intraocular inflammatory conditions, including diabetic macular edema (DME) and non-infectious uveitis such as Behçet’s disease (BD), Birdshot retinochoroidopathy (BSRC), HLA-B27-associated uveitis, and undifferentiated uveitis (UU).

**Results:**

A total of 77 subjects were included. mCRP levels were significantly elevated in BD, DME, and UU compared to controls (p = 0.014, p = 0.036, and p = 0.031, respectively). The mCRP/pCRP ratio was also significantly higher in DME and UU (p = 0.035 and p = 0.011, respectively). In addition, a strong positive correlation was observed between IL-6 and IL-1β (ρ = 0.638, p <0.0001). No significant differences in serum levels of pCRP, IL-6, or IL-1β were observed among the groups.

**Conclusions:**

These findings suggest that mCRP, rather than pCRP, may be a more specific systemic biomarker for certain intraocular inflammatory conditions. The involvement of the CRP axis and the strong correlation between IL-6 and IL-1β underscore the interaction of these key inflammatory mediators, providing further insight into the targeting of CRP axis for therapeutic purposes.

## 1 Introduction

The acute phase reactant, C-reactive protein (CRP) is a key regulator of the innate immune system and a sensitive biomarker of several inflammatory disorders. It has been traditionally linked to inflammatory conditions, including cardiovascular disease (1), autoimmune diseases (2), cancer (3) and COVID-19 (4). CRP is mainly synthesized in the liver under transcriptional control of interleukin-6 (IL-6) and, to a lesser extent, interleuquin-1β (IL-1β) and tumor necrosis factor-α and is typically found in plasma as a 115 kDa cyclic pentamer (pCRP) composed of five 23 kDa subunits (5). Under acidic conditions, oxidative stress or by means of bioactive lipids from the surface of activated or damaged cells, this pentameric conformation can dissociate into its 23 kDa monomeric subunits (mCRP) (6). This monomeric form, which represents the tissue-associated form of CRP, shows different biological properties and antigenicity, making it a more specific marker of localized inflammation and a key player in complement system activation, tissue damage, and the progression of chronic inflammation (7–9). *In vitro* and *in vivo* studies have demonstrated a broad spectrum of proinflammatory activities attributed to mCRP. These include recruitment of monocytes and lymphocytes, upregulation of proinflammatory cytokines such as CCL2 and IL-8, polarization of macrophages and T-cells towards a proinflammatory phenotype, and promotion of angiogenic processes (10, 11). In addition, mCRP plays a dual role in modulating the complement system by both recruiting complement proteins, such as C1q, to the surface of damaged cells, initiating complement activation and amplifying the inflammatory loop (9), and interacting with complement inhibitors like C4b-binding protein (C4BP) and complement factor H (FH), disrupting the local balance between complement activation and inhibition (7, 12).

Whether systemic mCRP can serve as an appropriate biomarker of ongoing local inflammation is a matter of debate. In the last years, some studies have determined systemic mCRP levels in different inflammatory disorders including cardiovascular disorders (13), autoimmune diseases (14), and age-related macular degeneration (15), a chronic degenerative disorder of the outer retina. Indeed, both CRP conformations, pCRP and mCRP, have been detected in the subretinal space (16). The specific contribution of local versus systemic CRP to ocular inflammation remains an area of ongoing investigation. It has been suggested that pCRP in the choroidal circulation may dissociate locally into mCRP, activating complement at the site and potentially contributing to retinal inflammation. Alternatively, mCRP may be primarily generated on the surface of activated choroidal endothelial cells, subsequently traversing the outer blood-retinal barrier and promoting a chronic inflammatory response within the retinal pigment epithelium (RPE)-Bruch’s membrane complex (17).

Non-infectious uveitis is a heterogeneous group of sight-threatening inflammatory conditions involving intraocular or chorioretinal tissues, suspected to be autoimmune or immune mediated (18). Non-infectious uveitis represents a collection of over 30 conditions that may present as isolated ocular disorders, such as Birdshot retinochoroidopathy (BSRC) or as manifestations of systemic syndromes such as Behçet’s disease (BD) or Vogt Koyanagi Harada syndrome. Nonetheless, many cases of noninfectious uveitis remain unclassifiable and are referred to as ‘undifferentiated’. In addition, macular edema stands for the leading cause of visual impairment in both diabetic retinopathy (19) and non-infectious uveitis (20), caused by blood-retinal barrier breakdown and increased vascular permeability, orchestrated by inflammatory mediators (21).

In this study, we aimed to evaluate the systemic levels of CRP axis, including pCRP, mCRP, IL-6 and IL-1β in various intraocular inflammatory conditions including diabetic macular edema (DME) and several types of non-infectious uveitis, comprising BD, BSRC, human leukocyte antigen (HLA)-B27-associated uveitis, and undifferentiated uveitis (UU). The findings could contribute to the understanding of the CRP axis in these pathologies from a systemic perspective which could offer novel therapeutic approaches for intraocular inflammation.

## 2 Methods

### 2.1 Study design, patients, and data collection

A retrospective observational study was conducted on 77 Caucasian patients with a variety of intraocular inflammatory disorders admitted to the Ophthalmology Department of Hospital Clinic of Barcelona, Spain, including BD (n=12), BSRC (n=10), UU (n=11), HLA-B27+ associated uveitis (n=16) and DME (n=12), and healthy control subjects (n=16). For patients with BD, HLA-B27+, BSRC, and UU, only those with active uveitis were included. Subjects were included between 2013 and 2017, and the study was approved by the local Institutional Review Board (Ethics and Clinical Investigation Committee, Hospital Clínic of Barcelona) and carried out according to the Declaration of Helsinki. All participants provided written informed consent to join the study. Data on age, sex, date of sample collection, current medication, and uveitis status (if applicable) were retrospectively collected for all patients in the study. The control group was geographically and socio-economically matched and consisted of 16 individuals with no history of cancer within the past 10 years, no immune-mediated disorders and not currently undergoing treatment with anti-inflammatory drugs, including corticosteroids, non-steroidal anti-inflammatory drugs, immunosuppressants, or biologic therapies.

### 2.2 Determination of pCRP, mCRP, IL-6 and IL-1β levels in serum samples

Serum samples were obtained from peripheral blood from venous puncture and stored at -80°C until use. pCRP was determined using a commercial ELISA kit specific for human CRP (high-sensitivity CRP, IBL International GmbH). IL-6 and IL-1β concentrations were determined using the ELISA DuoSet Human IL-6 and Human IL-1 beta/IL-1F2 kits (R&D Systems, Minneapolis, MN, USA), respectively. All tests were performed following the manufacturer’s instructions.

Serum mCRP was detected with an ELISA assay following the protocol described by Zhang et al. with some modifications (22). For this purpose, the mouse anti-human CRP monoclonal antibody CRP-8 (Sigma-Aldrich, C1688) was used as a capture antibody and immobilized at a dilution of 1:1,000 in coating buffer (10 mM sodium carbonate/bicarbonate, pH 9.6) overnight at 4°C. This commercially available monoclonal antibody is known to specifically bind to mCRP without cross-reacting with pCRP (23). The plates were washed three times, each for 2 minutes, using TBS, followed by blocking of non-specific binding sites with filtered 1% BSA-TBS for 1 hour at 37°C. Samples, diluted 1:20 in blocking buffer, were then added to the wells and incubated for 1 hour at 37°C. After repeating the washing steps, samples were treated with a sheep anti-human CRP polyclonal antibody (MBS223280, MyBioSource) at a dilution of 1:5,000 in blocking buffer for 1 hour at room temperature. Subsequently, an HRP-conjugated donkey anti-sheep IgG (Abcam), diluted 1:10,000 in blocking buffer, was applied. Signal detection was performed using a VersaMax Microplate Reader, and the optical density (OD) of each sample was calculated as OD450–OD570 nm.

A standard curve was generated by performing serial dilutions of mCRP (ranging from 0 to 100 ng/mL) prepared by urea-chelation of pCRP (Calbiochem) in a blocking buffer containing 1% BSA-TBS and reference diluted sera at a 1:20 ratio. Control experiments with purified pCRP at a concentration of 1 μg/mL produced only background signals, confirming the assay’s specificity for mCRP.

### 2.3 Statistical analysis

Descriptive statistics, including the median, minimum, and maximum values, were calculated for pCRP, mCRP, IL-6, IL-1β and the mCRP/pCRP ratio within each pathology group and controls. Additionally, the detection rate of mCRP, IL-6 and IL-1β was calculated for each group. Pairwise comparisons between pathology and control groups were performed using the Mann-Whitney U test (Wilcoxon rank-sum test) for each variable. P-values were adjusted for multiple comparisons using the Benjamini-Hochberg method to control the false discovery rate (FDR). Fisher’s exact test was used to compare the detection rates of mCRP, IL-6 and IL-1β across groups, with p-value adjustments for FDR. Spearman’s correlation coefficient was used to assess the relationship between variables. The age distribution and sex ratio were analyzed by reporting the median, range, and percentage of male and female participants for each group. Differences in mCRP levels by age and sex were examined using the Kruskal-Wallis test for age groups and the Mann-Whitney U test for sex-based comparisons. All statistical tests were two-tailed, with significance set at p < 0.05. Statistical analysis was conducted using R software (R Foundation for Statistical Computing, Vienna, Austria). Data visualization was conducted using the ggplot2 package to facilitate results interpretation.

## 3 Results

The study population consisted of 77 patients, including 12 with BD, 12 with DME, 16 with HLA-B27, 10 with BSRC, 11 with UU, and 16 healthy controls (**Table 1**). Control and DME subjects were significantly older than BD, HLA-B27, BSRC, and UU patients. No significant differences in sex distribution were observed across groups. Out of the 77 patients, data on medication treatment was available for 66 individuals, of whom 39 (59.09%) were undergoing some form of therapy, including corticosteroids, immunosuppressants, and antidiabetics.

**Table 1:** Descriptive analysis of control and intraocular inflammatory disorders.

Variable	Control (N=16)		Disease Cohorts					
			Combined (N=61)	BD (N=12)	DME (N=12)	HLAB-27 (N=16)	BSRC (N=10)	UU (N=11)
Age, median (min-max), years	70 (63-81)		47 (20-79)	**37.5 (20-47)**	75 (57-79)	**36 (33-69)**	**48.5 (30-63)**	**48 (31-66)**
Male (%)	31.3		52.6	66.7	50.0	62.5	20.0	54.5
Systemic treatment								
Yes, N		0	39	10	11	7	7	4
No, N		16	11	0	1	5	0	5
No data, N		0	11	2	0	4	3	2
Analytical Variables, median (min-max)								
pCRP, µg/ml		2.29 (0.35-7.17)	2.28 (0.19-20.5)	1.49 (0.19-20.5)	2.35 (0.44-5.49)	6.51 (0.18-16.6)	3.57 (0.37-15.61)	0.90 (0.12-6.06)
mCRP, ng/ml		10.0 (10-1335)	**277.1** **(10-5881)**	**424.1** **(10-1865)**	**292.7** **(10-5881)**	224.1 (10-1536)	139.7 (10-433)	**403.1** **(10-670)**
mCRP/pCRP ratio		0.01	**0.10**	0.2	**0.13**	0.02	0.06	**0.26**
mCRP detection rate, %, (N)		25.0 (4)	**75.4 (46)**	**83.3 (10)**	**75.0 (9)**	**68.7 (11)**	**70.0 (7)**	**81.8 (9)**
IL-6, pg/mL		0.74 (0.01-2318)	0.676 (0.01-1879)	0.82 (0.01-24.0)	0.835 (0.01-6.28)	3.24 (0.01-1879)	0.73 (0.01-92.0)	0.22 (0.01-1135)
IL-6 detection rate, %, (N)		75.0 (12)	70.5 (43)	50.0 (6)	91.7 (11)	87.5 (14)	60 (6)	54.5 (6)
IL-1β, pg/mL		0.01 (0.01-164)	0.01 (0.01-903)	0.01 (0.01-0.63)	0.01 (0.01-13.2)	0.01 (0.01-903)	0.01 (0.01-6.02)	0.01 (0.01-103)
IL-1β detection rate, %, (N)		25.0 (4)	18.0 (11)	16.7 (2)	8.3 (1)	31.3 (5)	10 (1)	18.2 (2)

Statistically significant values vs. control are highlighted in bold.

**Figure 1:**
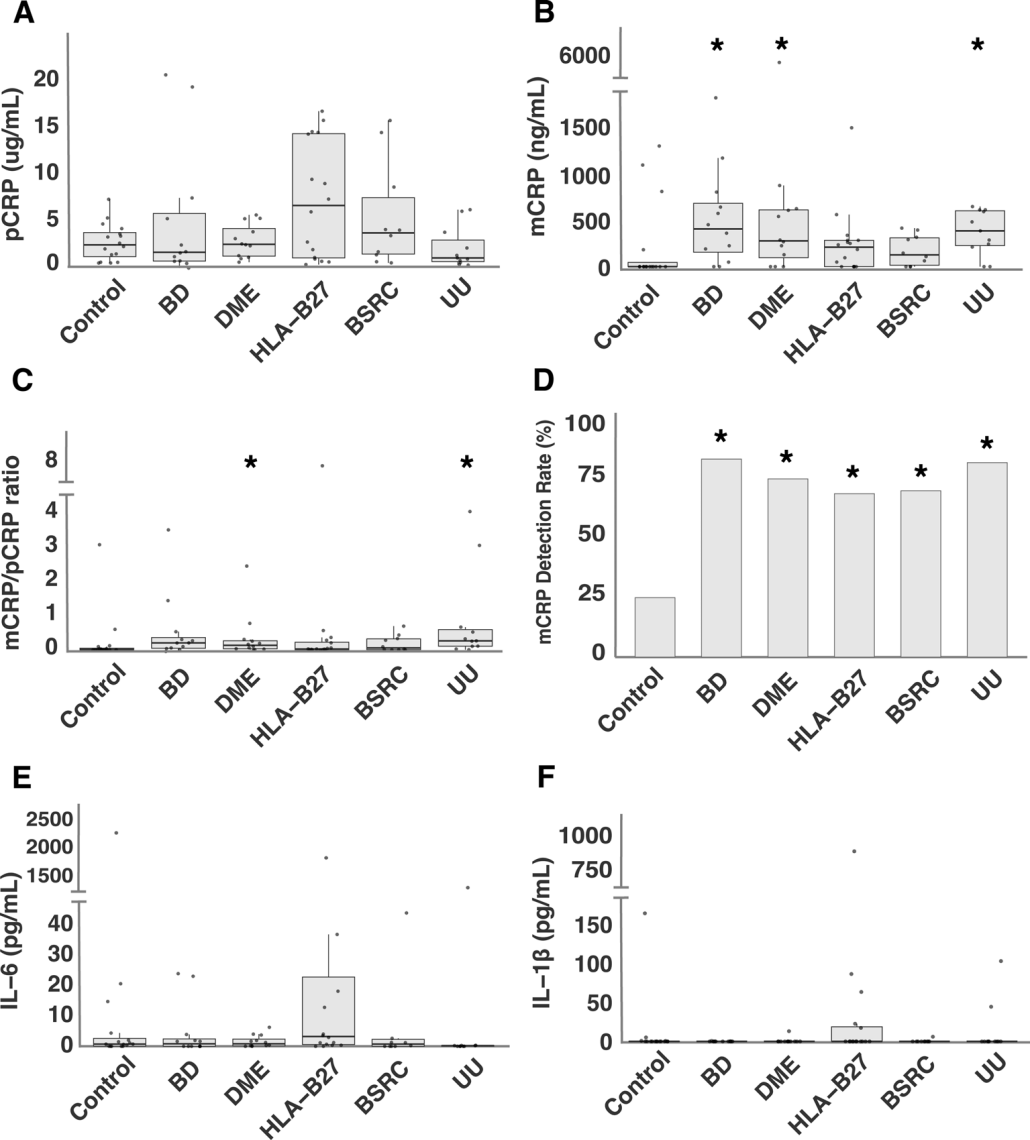
Systemic levels of CRP-axis in intraocular inflammatory conditions: **(A)** pCRP levels, **(B)** mCRP levels, **(C)** mCRP/pCRP ratio, **(D)** mCRP detection rate (%), **(E)** IL-6 levels, and **(F)** IL-1β. levels in intraocular inflammatory conditions and control group. *P<0.05 vs. control.

Serum levels of pCRP, measured by hsCRP ELISA, were similar across pathology and control groups, with all p-values exceeding 0.05. The distribution of pCRP concentrations across the study participants is shown in **Figure 1A**.

Compared to the control group, mCRP levels in BD, DME and UU were significantly higher. The adjusted p-values for the comparisons were as follows: BD *vs*. control (p = 0.014), DME *vs*. control (p = 0.036), UU *vs*. control (p = 0.031), while no significant differences were observed for HLA-B27-associated uveitis (p = 0.127) and BSRC (p = 0.290) (**Figure 1B**). Furthermore, when comparing mCRP/pCRP ratio between pathology groups and controls, DME and UU demonstrated a significantly different ratio compared to controls (p = 0.035 and p = 0.011, respectively) (**Figure 1C**). The detection rate of mCRP was significantly higher in all groups as compared to the control group. The adjusted p-values for the comparisons were as follows: BD *vs*. control (p = 0.006), DME *vs*. control (p = 0.020), HLA-B27-associated uveitis *vs*. control (p = 0.032), BSRC *vs*. control (p = 0.040), and UU *vs*. control (p = 0.006) (**Figure 1D**). No differences in mCRP levels were observed between males and females (p = 0.676). Similarly, mCRP levels were comparable across different age ranges (20-30, 31-40, 41-50, 51-60, 61-70, 71-80, 81+) as determined by a Kruskal-Wallis test (p = 0.988).

IL-6 was detected in 55 out of 77 samples (71.4%), while IL-1β was detected only in 15 out of 77 samples (19.5%). No significant differences were observed in systemic levels of IL-6 and IL-1β between pathology and control groups (all p-values > 0.05) (**Figures 1E, F**). Similarly, detection rates of IL-6 and IL-1β did not differ significantly across groups (all p-values > 0.05).

Spearman correlation analysis was conducted to evaluate the relationships between pCRP, mCRP, IL-6, and IL-1β within the disease group. A strong and statistically significant positive correlation was observed between IL-6 and IL-1β (ρ = 0.638, p <0.0001). Additionally, a positive trend was noted between pCRP and IL-6 (ρ = 0.219), suggesting a potential, though non-significant, relationship (p = 0.090). No significant correlations were found for the other correlations: pCRP *vs*. mCRP (p = 0.177), pCRP *vs*. IL-1β (p = 0.852), mCRP *vs*. IL-6 (p = 0.504), and mCRP *vs*. IL-1β (p = 0.639).

## 4 Discussion

This study aimed to analyze systemic mCRP and the overall status of the CRP pathway in a variety of intraocular inflammatory disorders, including DME and various types of non-infectious uveitis. We observed that systemic mCRP, but not pCRP, IL-1β, and IL-6, was detectable and significantly higher in some intraocular inflammatory disorders compared to healthy subjects.

The inflammatory profile of the included inflammatory intraocular conditions varies in terms of ocular localization, severity, and systemic involvement. In DME, which manifests as localized retinal inflammation, the condition is intrinsically linked to systemic metabolic disturbances, particularly the chronic low-grade inflammation associated with diabetes mellitus (24). Metabolic changes, such as elevated levels of advanced glycation end-products, drive oxidative stress and endothelial dysfunction, triggering an inflammatory cascade that disrupts both the blood–retinal barrier and the blood–aqueous barrier (21). This breakdown increases vascular permeability, leading to fluid accumulation in the retina and the development of macular edema. In parallel, non-infectious uveitis represents a diverse group of immune-mediated diseases, with varying degrees of systemic manifestations. BD is a systemic vasculitis that affects not only the eye but also skin, mucous membranes, and other organs, often leading to severe ocular inflammation and retinal vasculitis (25). The pathophysiology of BD is strongly influenced by the interaction between innate and adaptive immunity. Regarding HLA-B27-associated uveitis, it typically presents as recurrent, acute anterior uveitis and is frequently associated with systemic diseases such as ankylosing spondylitis, reactive arthritis, inflammatory bowel disease, and psoriatic arthritis (26). Nevertheless, it can also manifest as an isolated ocular disorder without systemic involvement. On the other hand, BSRC is strictly localized to the eye with no extraocular manifestations or systemic disease associations. This disease manifests as chronic, bilateral, posterior uveitis characterized by multiple white-creamy choroidal spots. HLA-A29 is strongly linked to BSCR, indicating that T-cells may play a central role in its pathogenesis (27). Finally, UU, characterized by diverse ocular inflammatory patterns and the absence of a clear systemic association, may result from immune system dysregulation (28). This could be due to underlying systemic diseases that are either undiagnosed or not sufficiently advanced to be classified, potentially influencing the inflammatory response (29). The inflammatory component of the included disorders, although through different mechanistical pathways, can result in detectable levels of mCRP in the systemic circulation and in altered levels of pCRP, IL-1β and IL-6.

Serum levels of pCRP might be expected to correlate with the presence of systemic inflammatory diseases, as pCRP is a marker of systemic inflammation that is often elevated in such conditions. However, in our study, despite higher median serum levels in certain conditions, no significant differences in pCRP levels were observed between any disease and control groups. Indeed, elevated circulating pCRP levels have been reported in active BD (30) and in a cohort of non-infectious uveitis (31). Such differences can be attributed to differences in medication regimens. Indeed, although non-statistically significant, we observed increased pCRP levels in patients with HLA-B27-associated uveitis (median 6.51 µg/ml) compared to the control group (median 2.29 µg/ml, p = 0.086). Previous studies have reported that pCRP alone may not be a reliable marker of disease activity in HLA-B27-associated uveitis and BD, as no correlation was found between CRP levels and the clinical presentation of uveitis (32). Instead, the CRP/albumin ratio was proposed as a more sensitive marker for identifying acute uveitis in patients with these conditions (31, 32).

While pCRP is routinely measured in clinical practice, mCRP represents the proinflammatory conformation released at sites of local inflammation (33). Upon dissociation, mCRP predominantly remains bound to cell membranes at these sites. Due to its insoluble nature, it is challenging to detect mCRP in serum. Some authors have detected circulating mCRP associated to microparticles (34) and also in serum using different experimental approaches, such as flow cytometry (13) and immunoassays (35–37). In our study, we adapted the ELISA protocol described by Zhang et al. (22), employing a capture antibody (clone CRP-8) that prevents cross-reactivity with pCRP. Noteworthy, we detected mCRP in 64.1% (50/78) of analyzed samples, with a detection rate significantly higher in all disease groups compared to the control group.

Systemic levels of mCRP have only been reported in a small number of disorders. Acute inflammatory disorders such as COVID-19 (38), myocardial infarction (37, 39), chronic obstructive pulmonary disease (36) or autoimmune disorders including systemic lupus erythematosus (14) and adult Still’s disease (35) have shown detectable levels of systemic mCRP. Instead, age-related macular degeneration which is associated with a milder systemic inflammation has shown low detection rates of systemic mCRP (15). Indeed, in our study we observed a significantly higher detection rate of mCRP in the pathology group compared to the control group (75% *vs*. 25%, p=0.016). Although, differences in mCRP detection rate can be attributed to methodological differences, it is also conceivable that systemic mCRP is only detectable upon certain inflammatory threshold. In our study, we included diseases such as BD and HLA-B27-associated uveitis, that are characterized by systemic involvement resulting in an inflammatory state, which may explain the higher mCRP detection rate. Nonetheless, we also included patients with BSCR, where the manifestations are confined to ocular structures, or in the case of UU, where no systemic manifestations have been clearly linked. This suggests that the elevated mCRP detection rate could be related not only to systemic manifestations but also to the development of ocular inflammation that somehow is eventually reflected in higher levels of circulating mCRP. Of note, when all disease groups were combined (n = 61) and compared to the control group, a significant increase in mCRP serum levels was observed (277.1 ng/ml *vs.* 10 ng/ml, p = 0.009). Subsequently, we evaluated each disease group individually against the control group and found that mCRP serum levels were significantly elevated in BD, DME, and UU groups, suggesting that mCRP may serve as a more specific marker of inflammation in these diseases. Notably, we did not observe neither age- nor sex-related differences in mCRP levels. Nevertheless, the lack of commercially available assays for measuring serum mCRP limits its potential translation to the clinical practice and restricts the comparability across studies due to methodological inconsistencies. The development of a standardized method to detect circulating mCRP would be highly beneficial to implement mCRP as a more specific biomarker of ongoing inflammation in certain conditions.

Because mCRP dissociates from pCRP under proinflammatory microenvironments we determined mCRP/pCRP as an additional measure of ongoing inflammation (ongoing CRP dissociation). When all disease groups were combined and compared to the control group, we observed a significantly higher mCRP/pCRP ratio in the pathology group (0.01 *vs.* 0.1, p = 0.037). Then, upon analyzing each disease individually, we observed that the mCRP/pCRP ratio was significantly higher in DME and UU groups, suggesting its potential role as a biomarker or distinguishing factor for these conditions.

In the present study, no significant differences were observed in serum levels of IL-6 and IL-1β between each disease group and the control group. For non-infectious uveitis, limited and heterogeneous findings have been reported regarding the systemic levels of IL-1β. Previous studies found no significant differences in serum levels of IL-1β in patients with BD (30). However, specific interleukin-1 gene polymorphisms have been associated with increased susceptibility to BD (40). In our cohort, the low detection rate of IL-1β (19.5%) might have limited our ability to identify significant differences. On the other hand, increased IL-6 levels have been reported in the serum of patients with active non-infectious uveitis (41), but more consistent findings have been observed in local ocular samples (42, 43). Indeed, tocilizumab, a neutralizing monoclonal antibody that targets the IL-6 receptor (IL-6R), has been reported to be effective for the treatment of uveitis and its associated macular edema (43, 44) and ongoing clinical trials are testing the efficacy of vamikibart, an intravitreal monoclonal antibody targeting IL-6 for the treatment of both, uveitic macular edema and DME (45).

A key finding of our study was the strong positive correlation between IL-6 and IL-1β (ρ = 0.638, p <0.0001) in our cohort. The robust relationship between IL-6 and IL-1β suggests a coordinated inflammatory response, where the activation of one cytokine may amplify the activity of the other, thereby contributing to the progression of inflammation. This interplay is likely mediated by the activation of the NLRP3 inflammasome, which responds to endogenous damage-associated molecular patterns (DAMPs) and triggers IL-1β activation. IL-1β then stimulates the cellular release of IL-6, which in turn promotes pCRP synthesis in hepatocytes (46). Nevertheless, in our study, we did not observe a significant correlation between IL-6 and pCRP levels (ρ = 0.219, p = 0.090). Also, mCRP levels were not associated neither with pCRP nor with IL-6 and IL-1β. Given that mCRP dissociates from pCRP one might expect a strong correlation between mCRP and pCRP. It could be that mCRP correlates with pCRP only above a certain threshold of systemic pCRP, sufficient to result in increased mCRP. Indeed, mCRP has been shown to correlate with pCRP in acute inflammatory disorders with high pCRP levels such as COVID-19 but not in AMD patients with low levels of pCRP. The lack of a linear relationship between mCRP and pCRP may be due to their distinct synthesis mechanisms. Although mCRP is mostly generated from pCRP dissociation in proinflammatory microenvironments (47), it can also be locally synthesized by macrophages and adipocytes (48, 49). It is also plausible that certain inflammatory ocular conditions provide the scenario for CRP dissociation whereas healthy subjects cannot provide the conditions for CRP dissociation, despite having similar levels of pCRP, which could explain the non-linear relationship between mCRP and pCRP levels, which can vary depending on the disease and patient population. Whether CRP dissociates locally at the site of ocular inflammation and is released into the systemic circulation (likely associated to microparticles) or it is generated in the circulation through binding to activated cells warrants further investigation that would certainly shed light on the dynamics of CRP dissociation under pathophysiological conditions. The lack of correlation between systemic CRP conformations and IL-1β or IL-6 may be attributed to the low levels of these cytokines detected in our cohort. All these mediators are interrelated, but it is unknown the exact timeline of this relationship in ocular inflammatory disorders and it is also likely that the local expression (either in aqueous humor or at the tissue level) are more strongly associated than the systemic levels.

The main limitations of this study include the small sample size for each disease group and the retrospective nature of the study that could introduce bias, as the findings may not fully represent a broader patient population. Nevertheless, by evaluating different pathologies together, we aim to standardize the results, allowing for meaningful comparisons across various groups. Additionally, though clinically active at the time of sample collection, the treatment of some patients with corticosteroids and immunosuppressants may have influenced CRP, IL-6 and IL-1β systemic levels. These factors should be considered when interpreting the results, and future studies with larger sample sizes and multi-center designs would be valuable to further explore these findings. Despite the limitations of our study, our results support the role of the IL-1β/IL-6/CRP axis in certain intraocular inflammatory disorders, with local and/or systemic involvement.

The potential to associate serum levels of mCRP with the development of local manifestations such as DME or certain forms of non-infectious uveitis could represent an important advance in evaluating disease progression and, even more so, a potential target for therapeutic purposes.

## Data Availability

The raw data supporting the conclusions of this article will be made available by the authors, without undue reservation.

## References

[1] Ridker PM Hennekens CH Buring JE Rifai N. C-reactive protein and other markers of inflammation in the prediction of cardiovascular disease in women. N Engl J Med. (2000) 342:1066–7. doi: 10.1056/NEJM200003233421202 10733371

[2] Caricchio R Gallucci S. Systemic lupus erythematosus and cytokine storm. Adv Exp Med Biol. (2024) 1448:355–64. doi: 10.1007/978-3-031-59815-9_24 39117826

[3] Hart PC Rajab IM Alebraheem M Potempa LA. C-reactive protein and cancer-diagnostic and therapeutic insights. Front Immunol. (2020) 11:595835. doi: 10.3389/FIMMU.2020.595835 33324413 PMC7727277

[4] Vogi V Haschka D Forer L Schwendinger S Petzer V Coassin S. Severe COVID-19 disease is associated with genetic factors affecting plasma ACE2 receptor and CRP concentrations. Sci Rep. (2025) 15(1):4708. doi: 10.1038/S41598-025-89306-4 39922945 PMC11807156

[5] Black S Kushner I Samols D. C-reactive protein. J Biol Chem. (2004) 279:48487–90. doi: 10.1074/jbc.R400025200 15337754

[6] Braig D Nero TL Koch HG Kaiser B Wang X Thiele JR. Transitional changes in the CRP structure lead to the exposure of proinflammatory binding sites. Nat Commun. (2017) 8:14188. doi: 10.1038/ncomms14188 28112148 PMC5264208

[7] Molins B Fuentes-Prior P Adán A Antón R Arostegui JI Yagüe J. Complement factor H binding of monomeric C-reactive protein downregulates proinflammatory activity and is impaired with at risk polymorphic CFH variants. Sci Rep. (2016) 6:22889. doi: 10.1038/srep22889 26961257 PMC4785391

[8] Molins B Peña E Vilahur G Mendieta C Slevin M Badimon L. C-reactive protein isoforms differ in their effects on thrombus growth. Arterioscler Thromb Vasc Biol. (2008) 28:2239–46. doi: 10.1161/ATVBAHA.108.174359 18787187

[9] Thiele JR Habersberger J Braig D Schmidt Y Goerendt K Maurer V. Dissociation of pentameric to monomeric C-reactive protein localizes and aggravates inflammation: *In vivo* proof of a powerful proinflammatory mechanism and a new anti-inflammatory strategy. Circulation. (2014) 130:35–50. doi: 10.1161/CIRCULATIONAHA.113.007124 24982116

[10] McFadyen JD Kiefer J Braig D Loseff-Silver J Potempa LA Eisenhardt SU. Dissociation of C-reactive protein localizes and amplifies inflammation: Evidence for a direct biological role of C-reactive protein and its conformational changes. Front Immunol. (2018) 9:1351. doi: 10.3389/fimmu.2018.01351 29946323 PMC6005900

[11] Molins B Pascual A Méndez Llorenç V Zarranz-Ventura J Mesquida M. C-reactive protein isoforms differentially affect outer blood-retinal barrier integrity and function. Am J Physiol - Cell Physiol. (2017) 312:C244–53. doi: 10.1152/ajpcell.00057.2016 28003224

[12] Lauer N Mihlan M Hartmann A Schlötzer-Schrehardt U Keilhauer C Scholl HPN. Complement regulation at necrotic cell lesions is impaired by the age-related macular degeneration-associated factor-H his 402 risk variant. J Immunol. (2011) 187:4374–83. doi: 10.4049/jimmunol.1002488 21930971

[13] Melnikov I Kozlov S Pogorelova O Tripoten M Khamchieva L Saburova O. The monomeric C-reactive protein level is associated with the increase in carotid plaque number in patients with subclinical carotid atherosclerosis. Front Cardiovasc Med. (2022) 9:968267. doi: 10.3389/FCVM.2022.968267 35935662 PMC9353581

[14] Karlsson J Wetterö J Weiner M Rönnelid J Fernandez-Botran R Sjöwall C. Associations of C-reactive protein isoforms with systemic lupus erythematosus phenotypes and disease activity. Arthritis Res Ther. (2022) 24(1):139. doi: 10.1186/S13075-022-02831-9 35690780 PMC9188243

[15] Giralt L Figueras-Roca M Eguileor BDL Romero B Zarranz-Ventura J Alforja S. C-reactive protein-complement factor H axis as a biomarker of activity in early and intermediate age-related macular degeneration. Front Immunol. (2024) 15:1330913. doi: 10.3389/FIMMU.2024.1330913 38633250 PMC11021604

[16] Mullins RF Russell SR Anderson DH Hageman GS. Drusen associated with aging and age-related macular degeneration contain proteins common to extracellular deposits associated with atherosclerosis, elastosis, amyloidosis, and dense deposit disease. FASEB J. (2000) 14:835–46. doi: 10.1096/fasebj.14.7.835 10783137

[17] Romero-Vázquez S Adán A Figueras-Roca M Llorenç V Slevin M Vilahur G. Activation of C-reactive protein proinflammatory phenotype in the blood retinal barrier *in vitro*: Implications for age-related macular degeneration. Aging (Albany NY). (2020) 12:13905–23. doi: 10.18632/aging.103655 PMC7425453 32673285

[18] Nussenblatt RB. The natural history of uveitis. Int Ophthalmol. (1990) 14:303–8. doi: 10.1007/BF00163549 2249907

[19] Antonetti DA Klein R Gardner T. Mechanims of disease: diabetic retinopathy. N Engl J Med. (2012) 366:1227–39. doi: 10.1056/NEJMra1005073 22455417

[20] Lardenoye CWTA van Kooij B Rothova A. Impact of macular edema on visual acuity in uveitis. Ophthalmology. (2006) 113:1446–9. doi: 10.1016/J.OPHTHA.2006.03.027 16877081

[21] Daruich A Matet A Moulin A Kowalczuk L Nicolas M Sellam A. Mechanisms of macular edema: Beyond the surface. Prog Retin Eye Res. (2017) 63:20–68. doi: 10.1016/J.PRETEYERES.2017.10.006 29126927

[22] Zhang L Li HY Li W Shen ZY Wang YD Ji SR. An ELISA assay for quantifying monomeric C-reactive protein in plasma. Front Immunol. (2018) 9:511. doi: 10.3389/FIMMU.2018.00511 29593741 PMC5857914

[23] Schwedler SB Guderian F Dämmrich J Potempa LA Wanner C. Tubular staining of modified C-reactive protein in diabetic chronic kidney disease. Nephrol Dial Transplant. (2003) 18:2300–7. doi: 10.1093/NDT/GFG407 14551357

[24] Das A McGuire PG Rangasamy S. Diabetic macular edema: pathophysiology and novel therapeutic targets. Ophthalmology. (2015) 122:1375–94. doi: 10.1016/j.ophtha.2015.03.024 25935789

[25] Mesquida M Molins B Llorenç V Hernández MV Espinosa G Dick AD. Current and future treatments for Behçet’s uveitis: road to remission. Int Ophthalmol. (2014) 34:365–81. doi: 10.1007/S10792-013-9788-5 23729309

[26] Wakefield D Clarke D McCluskey P. Recent developments in HLA B27 anterior uveitis. Front Immunol. (2021) 11:608134. doi: 10.3389/FIMMU.2020.608134 33469457 PMC7813675

[27] Nussenblatt RB Mittal KK Ryan S Green WR Maumenee AE. Birdshot retinochoroidopathy associated with HLA-A29 antigen and immune responsiveness to retinal S-antigen. Am J Ophthalmol. (1982) 94:147–58. doi: 10.1016/0002-9394(82)90069-1 6956239

[28] Llorenç V Mesquida M Sainz de la Maza M Keller J Molins B Espinosa G. Epidemiology of uveitis in a Western urban multiethnic population. The challenge of globalization. Acta Ophthalmol. (2015) 93:561–7. doi: 10.1111/AOS.12675 25683136

[29] Choi RY Rivera-Grana E Rosenbaum JT. Reclassifying idiopathic uveitis: lessons from a tertiary uveitis center. Am J Ophthalmol. (2019) 198:193–9. doi: 10.1016/J.AJO.2018.10.018 PMC6349540 30352197

[30] Mesquida M Molins B Llorenç V Sainz de la Maza M Hernandez MV Espinosa G. Proinflammatory cytokines and C-reactive protein in uveitis associated with Behçet’s disease. Mediators Inflammation. (2014) 2014:396204. doi: 10.1155/2014/396204 PMC4068062 24994946

[31] Bozkurt E Muhafiz E Sengul D Uçak T Atum M. Can the CRP/albumin ratio be used as a new indicator of activation in patients with uveitis? Ocul Immunol Inflammation. (2021) 29:1017–22. doi: 10.1080/09273948.2020.1714061 32125910

[32] Kim M Park YG Park YH. C-reactive protein/albumin ratio as an indicator of disease activity in Behçet’s disease and human leukocyte antigen-B27-associated uveitis. Graefes Arch Clin Exp Ophthalmol. (2021) 259:1985–92. doi: 10.1007/S00417-021-05207-Y 33929591

[33] Wu Y Potempa LA El Kebir D Filep JG. C-reactive protein and inflammation: conformational changes affect function. Biol Chem. (2015) 396:1181–97. doi: 10.1515/HSZ-2015-0149 26040008

[34] Crawford JR Trial JA Nambi V Hoogeveen RC Taffet GE Entman ML. Plasma levels of endothelial microparticles bearing monomeric C-reactive protein are increased in peripheral artery disease. J Cardiovasc Transl Res. (2016) 9:184–93. doi: 10.1007/S12265-016-9678-0 PMC4874871 26891844

[35] Fujita C Sakurai Y Yasuda Y Homma R Huang C-L Fujita M. mCRP as a biomarker of adult-onset still’s disease: quantification of mCRP by ELISA. Front Immunol. (2022) 0:938173. doi: 10.3389/FIMMU.2022.938173 PMC9284222 35844576

[36] Munuswamy R De BJ Burtin C Derave W Aumann J MA S. Monomeric CRP is elevated in patients with COPD compared to non-COPD control persons. J Inflammation Res. (2021) 14:4503–7. doi: 10.2147/JIR.S320659 PMC8434905 34522118

[37] Wang J Tang B Liu X Wu X Wang H Xu D. Increased monomeric CRP levels in acute myocardial infarction: a possible new and specific biomarker for diagnosis and severity assessment of disease. Atherosclerosis. (2015) 239:343–9. doi: 10.1016/J.ATHEROSCLEROSIS.2015.01.024 25682033

[38] Molins B Figueras-Roca M Valero O Llorenç V Romero-Vázquez S Sibila O. C-reactive protein isoforms as prognostic markers of COVID-19 severity. Front Immunol. (2023) 13:1105343. doi: 10.3389/FIMMU.2022.1105343 36741367 PMC9893772

[39] Habersberger J Strang F Scheichl A Htun N Bassler N Merivirta RM. Circulating microparticles generate and transport monomeric C-reactive protein in patients with myocardial infarction. Cardiovasc Res. (2012) 96:64–72. doi: 10.1093/CVR/CVS237 22798388

[40] Karasneh J Hajeer AH Barrett J Ollier WER Thornhill M Gul A. Association of specific interleukin 1 gene cluster polymorphisms with increased susceptibility for Behcet’s disease. Rheumatol (Oxford). (2003) 42:860–4. doi: 10.1093/RHEUMATOLOGY/KEG232R 12730545

[41] Kramer M Monselise Y Bahar I Cohen Y Weinberger D Goldenberg-Cohen N. Serum cytokine levels in active uveitis and remission. Curr Eye Res. (2007) 32:669–75. doi: 10.1080/02713680701523147 17852191

[42] Perez VL Papaliodis GN Chu D Anzaar F Christen W Foster CS. Elevated levels of interleukin 6 in the vitreous fluid of patients with pars planitis and posterior uveitis: the Massachusetts eye & ear experience and review of previous studies. Ocul Immunol Inflammation. (2004) 12:205–14. doi: 10.1080/092739490500282 15385196

[43] Mesquida M Molins B Llorenç V de la Maza MS Adán A. Targeting interleukin-6 in autoimmune uveitis. Autoimmun Rev. (2017) 16:1079–89. doi: 10.1016/J.AUTREV.2017.08.002 28778705

[44] Mesquida M Molins B Llorenç V Sainz de la Maza M Adán A. Long-term effects of tocilizumab therapy for refractory uveitis-related macular edema. Ophthalmology. (2014) 121:2380–6. doi: 10.1016/J.OPHTHA.2014.06.050 25204610

[45] Roche H-L. Study Details | A Study to Investigate Vamikibart (RO7200220) in Diabetic Macular Edema. ClinicalTrials.gov. (2021). Available online at: https://clinicaltrials.gov/study/NCT05151731.

[46] Libby P. Targeting inflammatory pathways in cardiovascular disease: the inflammasome, interleukin-1, interleukin-6 and beyond. Cells. (2021) 10(4):951. doi: 10.3390/CELLS10040951 33924019 PMC8073599

[47] Ji S-R Wu Y Zhu L Potempa LA Sheng F-L Lu W. Cell membranes and liposomes dissociate C-reactive protein (CRP) to form a new, biologically active structural intermediate: mCRP(m). FASEB J. (2007) 21:284–94. doi: 10.1096/fj.06-6722com 17116742

[48] Calabro P Chang DW Willerson JT Yeh ETH. Release of C-reactive protein in response to inflammatory cytokines by human adipocytes: linking obesity to vascular inflammation. J Am Coll Cardiol. (2005) 46:1112–3. doi: 10.1016/J.JACC.2005.06.017 16168299

[49] Ciubotaru I Potempa LA Wander RC. Production of modified C-reactive protein in U937-derived macrophages. Exp Biol Med. (2005) 230:762–70. doi: 10.1177/153537020523001010 16246904

